# MedFusionT5: Cross-Modal Attention Boosts Semantic Quality and Reduces Hallucinations in Dental AI

**DOI:** 10.1016/j.identj.2025.109404

**Published:** 2026-03-01

**Authors:** Hamida Abdaoui, Sabri Barbaria, Ismail Dergaa, Halil İbrahim Ceylan, Nicola Luigi Bragazzi, Andrea de Giorgio, Ridha Ben Salah, Hanene Boussi Rahmouni

**Affiliations:** a Laboratory of Biophysics and Medical Technologies, Higher Institute of Medical Technologies of Tunis (ISTMT), University of Tunis El Manar, Tunis, Tunisia; b High Institute of Sport and Physical Education of Ksar Said, University of Manouba, Manouba, Tunisia; c Physical Education of Sports Teaching Department, Faculty of Sports Sciences, Atatürk University, Erzurum, Türkiye; d Department of Mathematics and Statistics, Laboratory for Industrial and Applied Mathematics (LIAM), York University, Toronto, Ontario, Canada; e Department of Clinical Pharmacy, Saarland University, Saarbrücken, Germany; f Artificial Engineering, Naples, Italy; g The Computer Science Research Centre, the University of the West of England, Bristol, UK

**Keywords:** Medical report generation, Multimodal alignment, Dental AI, Hallucination control

## Abstract

**Introduction and aims:**

Automated dental report generation faces significant challenges in multimodal fusion, often resulting in suboptimal semantic quality and risks of hallucination, where AI generates clinically unsupported content. Current approaches that rely on simple feature concatenation or bidirectional attention mechanisms fail to effectively capture visual-textual relationships in medical imaging. This study aims to develop MedFusionT5, a unidirectional cross-modal alignment framework that (1) achieves superior clinical report quality through focused attention between visual patches and clinical text representations, and (2) ensures exceptional factual consistency by minimising hallucination rates.

**Methods:**

We implemented a novel architecture that integrates vision transformer (ViT) for patch-based visual feature extraction with Bio_ClinicalBERT for clinical text encoding. The core innovation is a unidirectional multihead attention alignment module that selectively maps textual embeddings to relevant visual patches before multimodal fusion. A T5-base decoder then generates diagnostic reports from the aligned representations. We evaluated performance on 700 dental panoramic radiographs using comprehensive metrics, including BLEU, ROUGE, CIDEr, clinical precision/recall, and specialised hallucination analysis, comparing against both concatenation and coattention baselines.

**Results:**

MedFusionT5 demonstrated superior performance across all evaluated metrics. Compared to the coattention baseline, CIDEr increased by 122% (5.65 vs 2.54) and by 320% over simple concatenation. BLEU-4 reached 0.865, outperforming both baselines, while maintaining the lowest hallucination rate at 2.42% (39% reduction vs coattention, 46% vs concatenation). The model achieved an optimal balance between precision (0.982) and recall (0.923), with 90% of reports exhibiting near-zero hallucination. Notably, MedFusionT5 showed consistent quality independent of report length (r = −0.022), unlike coattention's length-dependent performance (r = +0.795).

**Conclusion:**

MedFusionT5 establishes a new state-of-the-art in automated dental report generation, demonstrating that unidirectional cross-modal alignment achieves superior semantic quality and clinical precision while minimising hallucinations. This work identifies unidirectional attention as the optimal alignment strategy for medical AI, providing a foundation for trustworthy clinical deployment where both accuracy and reliability are paramount.

## Introduction

The automated generation of diagnostic reports from medical images is a critical challenge at the intersection of computer vision and natural language processing (NLP). The global shortage of radiologists creates substantial diagnostic delays in diverse health care settings.^1^^,^^2^ Rural hospitals struggle to provide timely interpretations without on-site specialists, with studies reporting average wait times exceeding 48 hours for nonurgent cases.^3^ Emergency departments require rapid preliminary reads for acute cases in which treatment decisions cannot wait hours for formal reports.^4^^,^^5^ Military field hospitals operate with limited diagnostic expertise in combat zones, creating critical gaps in trauma management.^6^ Developing nations face severe shortages of trained radiologists, with some countries reporting ratios below one radiologist *per* million population compared to 12 *per* million in high-income countries.^7^^,^^8^ Telemedicine initiatives can expand access but depend on asynchronous reporting, which introduces delays ranging from several hours to days.^9^^,^^10^ After-hours care suffers from limited specialist availability, forcing clinicians to defer diagnostic decisions until morning rounds.^11^^,^^12^

Dental panoramic radiographs exemplify these challenges in the following ways. These images allow dentists to identify mandibular fractures, periapical infections,^13^ impacted teeth, and temporomandibular joint disorders that require prompt intervention.^14^^,^^15^ Athletic injuries during evening competitions,^16^ trauma cases arriving at night,^17^ and dental emergencies in underserved regions^18^ all require immediate interpretation. Current workflows require images to be queued for specialist review, introducing delays that compromise care quality. For sports teams travelling internationally, rural clinics without dental radiologists, military units in deployment, and emergency departments managing multiple trauma patients simultaneously, automated preliminary reporting systems could substantially enhance diagnostic throughput while maintaining clinical accuracy.^19^^,^^20^ In this context, AI can play a pivotal role by providing rapid, reliable initial assessments of dental radiographs, thus reducing delays and enabling health care providers to deliver timely interventions, especially in areas with limited access to specialists.^21^^,^^22^

Recent transformer-based architectures have demonstrated promising results in medical image captioning^23^^,^^24^; however, fundamental limitations persist. Current state-of-the-art approaches predominantly rely on feature concatenation, where visual features from convolutional encoders are combined with textual embeddings before decoding.^25^^,^^26^ This fusion strategy fails to model the semantic correspondences between specific radiological findings and their clinical descriptions. Performance has plateaued, with recent systems showing incremental improvements of 2% to 5% annually across standard benchmarks.^27^^,^^28^ More critically, the absence of explicit grounding mechanisms allows language decoders to generate plausible but factually incorrect content, a phenomenon known as hallucination, which poses serious risks in clinical deployment.^29^^,^^30^

Although cross-attention mechanisms have proven effective in general vision-language tasks,^31^^,^^32^ their application in medical report generation remains underexplored. Existing medical AI systems, such as CheXbert and NeuroBERT, primarily focus on extracting structured information from existing reports rather than generating comprehensive diagnostic narratives.^33^^,^^34^ The few studies that have attempted end-to-end generation have not systematically investigated how different alignment strategies affect both semantic quality and factual consistency.^35^^,^^36^

While several recent works have explored cross-attention mechanisms for medical report generation, such as R2GenCSR^37^ with its context retrieval approach, the Cross-Modal Augmented Transformer^38^ with its ‘locate then generate’ paradigm, and MedVAG^39^ integrating multiple attention mechanisms, these methods have primarily focused on chest X-ray analysis. Our work fundamentally addresses these gaps through MedFusionT5, an advanced framework that utilises ViT for patch-based visual feature extraction and employs explicit multihead cross-attention to align these rich spatial features with clinical textual embeddings from Bio_ClinicalBERT before T5-based decoding. Unlike existing approaches that use bidirectional attention mechanisms, our framework introduces targeted unidirectional alignment specifically designed for dental panoramic radiography, which presents unique challenges in anatomical complexity and specialised terminology. We validate this approach through a comprehensive evaluation against both simple concatenation and cross-stream coattention baselines on 700 dental panoramic radiographs, demonstrating that our method's fine-grained visual-textual integration significantly enhances semantic coherence while addressing the critical challenge of scarce public dental panoramic datasets.

## Related work

### Medical report generation

Early approaches to medical report generation employed template-based methods and retrieval systems that matched images with existing reports.^40^^,^^41^ The advent of encoder-decoder architectures has enabled more flexible generation, with convolutional neural networks encoding images and recurrent networks decoding text.^42^ Attention mechanisms improved performance by allowing decoders to focus on relevant image regions,^43^ but these methods still struggled with clinical coherence and terminology accuracy.

As previously mentioned, transformer-based models have marked a significant advance. BERT-based systems, such as CheXbert, have demonstrated strong performance in labelling chest X-ray findings from existing reports,^33^ whereas T5 and VL-T5 have shown promise for generative tasks.^44^^,^^45^ However, these models typically address text-to-text transformation or general image captioning rather than the specialised requirements of medical reporting, where both domain-specific terminology and precise visual grounding are essential for accuracy and clarity.

### Multimodal fusion strategies

The question of how to effectively combine visual and textual information remains central to the field of vision-language research. Concatenation approaches, in which features are simply combined before decoding, offer computational simplicity but lack the ability to model fine-grained correspondences.^46^ Coattention mechanisms allow bidirectional interactions between modalities,^39^^,^^47^ whereas cross-attention enables queries from one modality to attend to another.^48^

In medical imaging, Chen et al^49^ proposed cross-modal memory networks for radiology reports, achieving modest improvements over concatenation. However, their approach focused on chest radiographs and did not investigate hallucination rates or provide a systematic comparison of fusion strategies. Our work extends this direction by implementing dedicated alignment modules and providing a comprehensive evaluation, including factual consistency metrics.

### Hallucination in medical AI

Hallucination, the generation of plausible but unsupported content, has emerged as a critical concern in large language models (LLMs).^29^^,^^50^ In medical contexts, where factual accuracy directly impacts patient safety, even minor hallucinations are unacceptable.^51^ Recent studies have proposed various detection methods, including embedding-based similarity measures and retrieval-augmented generation (RAG)^52^^,^^53^; however, the systematic measurement of hallucination rates in medical report generation remains rare.

To our knowledge, no prior study has quantified hallucination rates in dental radiology report generation or investigated whether architectural choices, such as alignment mechanisms, affect factual consistency. Our study addresses this gap by providing a comprehensive hallucination analysis alongside traditional NLP metrics.

## Methods

### Dataset and preprocessing

We utilised 700 dental panoramic radiographs with corresponding diagnostic reports from the OpenI database, a publicly accessible repository maintained by the U.S. National Library of Medicine containing de-identified biomedical images and associated clinical documentation. Each radiograph underwent preprocessing, including duplicate removal and cropping of nondiagnostic regions. The images were resized to 224 × 224 pixels and normalised to match the input requirements of the ViT encoder.

Reports were cleaned to remove artefacts from optical character recognition and were standardised for terminology consistency. The dataset was split into training (560 images, 80%), validation (70 images, 10%), and test (70 images, 10%) sets. Data augmentation during training included random horizontal flips, rotations (±10°), colour jittering (brightness ± 0.2, contrast ±0.2), and random resized cropping (scale 0.8-1.0) to improve model generalisation.

### Architecture overview

**Fig. 1:**
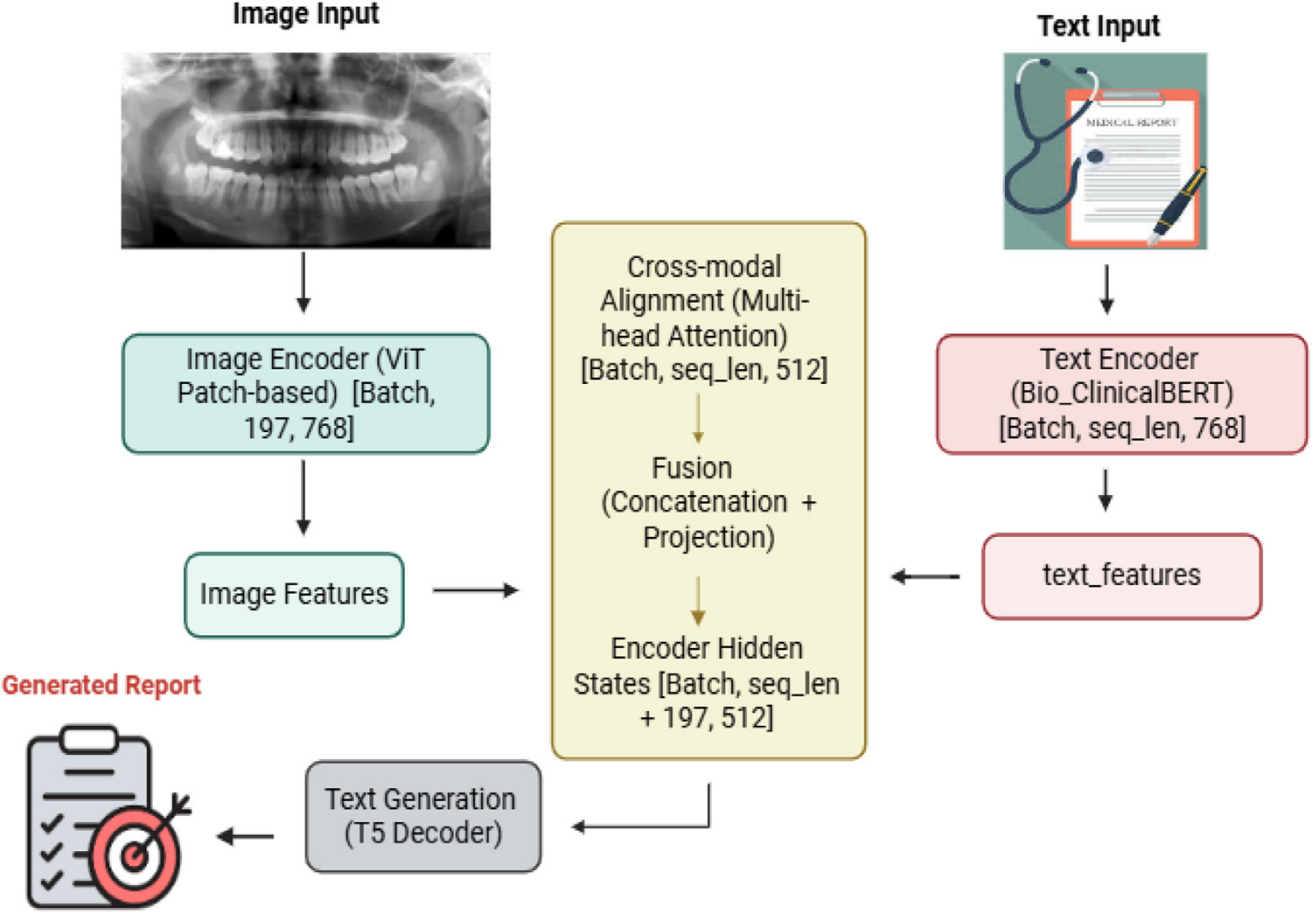
A schematic overview of the MedFusionT5 framework for automated medical report generation. The architecture consists of three core components: (1) patch-based visual encoding using vision transformer (ViT), (2) clinical textual encoding via Bio_ClinicalBERT with multihead cross-attention alignment for explicit visual-textual correspondence, and (3) T5-based report generation from the fused cross-modal representations.

Figure 1 illustrates the MedFusionT5 architecture, which comprises three main components: visual encoding, textual encoding with cross-modal alignment, and report generation.

#### Visual encoder

We utilise ViT-Base pre-trained on ImageNet-21K for visual feature extraction.^54^ The encoder processes input images (224 × 224 pixels) by dividing them into 16 × 16 patches, resulting in 196 patch embeddings along with a [CLS] token. The transformer architecture then outputs a sequence of 197 feature vectors, each with a dimension of 768, formulated as (Equation 1):

(1) $$Visual\;Features=ViT\left(x\right)\in R^{197\;\times \;768}$$

Where (x) represents the input image, this patch-based approach maintains spatial integrity and facilitates detailed cross-modal alignment with clinical textual representations. It eliminates the need for additional pooling or flattening operations that are typically required in CNN-based encoders.

#### Textual encoder

Reference reports were tokenised (maximum 512 tokens) and encoded using Bio_ClinicalBERT,^55^ which was pretrained on biomedical and clinical texts.

This produces contextual embeddings $H\in R^{B\times T\times \;768}$ (Equation 2), where B is the batch size, *T* is the sequence length, and 768 is BERT's output dimension. These embeddings are projected to 512 dimensions via a trainable matrix $W_{proj}\in R^{768\;\times \;512}$ for alignment (Equation 3).

(2) H=BERT(input_ids,attentionmask)

(3) $$text\_features=H.W_{proj}$$

#### Cross-modal alignment module

This module achieves fine-grained alignment between clinical text and visual patches using multihead cross-attention (Equation 4). In this process, the projected text embeddings act as queries, while the visual patch embeddings function as keys and values. The alignment is formulated as follows:

(4) aligned_text,attn_weights=MultiHeadAttention(Q=text_features,K=V=img_patches)

where:

$text\_features\in R^{B\times T\times \;512}$: projected text embeddings

$img\_patches\in R^{B\times \;197\;\times \;768}$: visual patch embeddings

$aligned\_text\in R^{B\times T\times \;512}$: aligned textual representations

attn_weights: attention patterns between text and image patches

The multihead attention employs 8 heads with a dimension $d_{k}=d_{v}=64$ *per* head, followed by layer normalisation with residual connection (Equation 5):

(5) output=LayerNorm(text_features+Dropout(aligned_text))

This enables the model to capture diverse visual-textual relationships while maintaining training stability through residual connections.

#### Report generator

The aligned text representations are concatenated directly with the visual patch embeddings along the sequence dimension to form the encoder hidden states (Equation 6). The encoder attention mask is accordingly extended to include both text and visual tokens (Equation 7). The T5-base model then generates the report autoregressively, conditioned on these fused representations (Equation 8), using its encoder-decoder architecture.

(6) encoder_hidden_states=Concat(aligned_text,img_patches)

(7) $$\text{encoder}\_\text{attention}\_\text{mask}=\text{Concat}\left(\text{attention}\_\text{mask},R^{B\times \;197}\right)$$

(8) generated_report=T5_Decoder(encoder_hidden_states,encoder_attention_mask)

### Training configuration

**Fig. 2:**
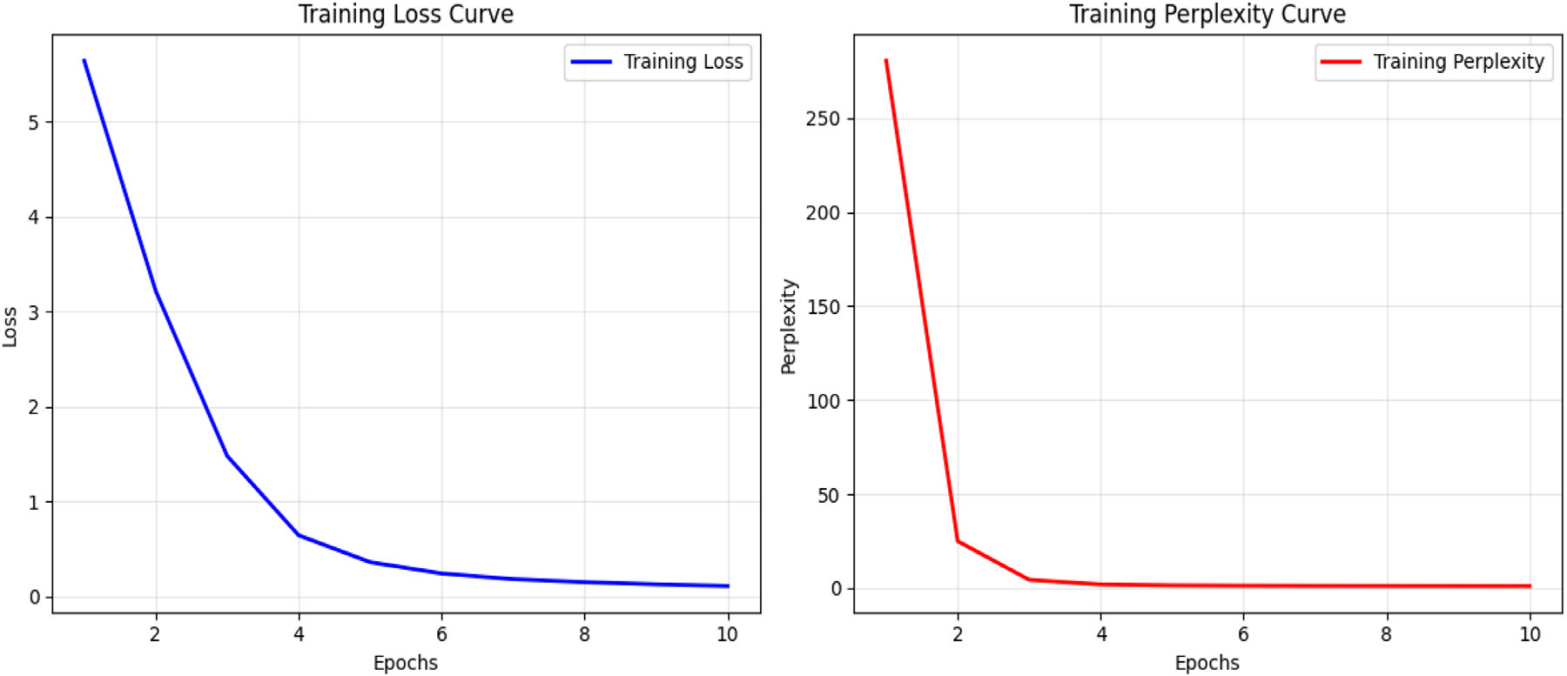
The training loss curve and the training perplexity curve both illustrate the model's quick convergence. The loss curve decreases rapidly early in training and stabilises around epoch 4, indicating effective learning. Similarly, the perplexity curve drops sharply in the first few epochs, reflecting the model's ability to reduce prediction uncertainty as training progresses.

Table 1 summarises the experimental parameters used. Training utilised a batch size of 8, the Adam optimiser with a learning rate of 5 × 10⁻⁵, and ran for 10 epochs. Figure 2 illustrates the training loss curve and the training perplexity curve.

**Table 1:** Model configuration and training parameters.

Component	Parameter	Value
Visual encoder	Model	Vision Transformer (ViT-Base)
	Pretraining dataset	ImageNet-21K
	Input size	224 × 224
	Patch size	16 × 16
	Number of patches	197 (196 + [CLS])
	Feature dimension	768
Text encoder	Model	Bio_ClinicalBERT
	Pretrained model	emilyalsentzer/Bio_ClinicalBERT
	Max token length	512
	Embedding dimension	768
	Projection dimension	512 (Linear: 768→512)
Alignment module	Attention type	Multihead Cross-Attention
	Number of heads	8
	Embedding dimension	512
	Normalisation	LayerNorm + Residual
	Dropout rate	0.1
Report decoder	Model	T5-base
	Encoder/decoder layers	12
	Attention heads	12
	Embedding dimension	768
	Feed-forward network dimension	3072
	Vocabulary size	32,128
Training	Optimiser	Adam
	Learning rate	5 × 10⁻⁵
	Batch size	8
	Epochs	10
	Loss function	Cross-Entropy
Sampling method	Decoding strategy	Beam search
	Number of beams	5
	Max output length	64 tokens

### Baseline comparison

To evaluate the effectiveness of our cross-modal alignment, we compared it to two baselines: (1) a simple concatenation approach that directly combines visual and textual features, and (2) a coattention model that employs cross-stream attention. All three models used the same encoders (ViT, Bio_ClinicalBERT) and decoders (T5-base), differing only in their fusion mechanisms.

### Evaluation metrics

We employ a comprehensive evaluation spanning three dimensions:

#### Lexical quality

Bilingual evaluation understudy (BLEU) measures n-gram overlap with reference texts.^56^ Metric for Evaluation of Translation with Explicit Ordering (METEOR) extends BLEU with stemming and synonym matching.^57^ The Jaccard similarity quantifies the set overlap.^58^

#### Semantic quality

Recall-Oriented Understudy for Gisting Evaluation (ROUGE) evaluates recall-oriented n-grams and sequence matching.^59^ Consensus-based Image Description Evaluation (CIDEr) measures consensus-based similarity using TF-IDF weighted n-grams and is particularly sensitive to clinically distinctive phrases.^60^

#### Clinical efficacy

Token-level precision, recall, and F1 scores were used to assess whether the generated reports captured the essential clinical content.^61^

#### Factual consistency

We quantify hallucination rates by comparing generated reports against reference texts and identifying content unsupported by visual evidence. Reports with <2% unsupported content are classified as having near-zero hallucinations.

## Results

### Overall performance comparison

Table 2 presents comprehensive evaluation metrics comparing MedFusionT5 with the concatenation and coattention baselines.

**Table 2:** Performance comparison between concatenation baseline and MedFusionT5.

Metric	Baseline (Concatenation)	Baseline (coattention)	MedFusionT5 (our model)
Lexical precision			
BLEU-1	0.7607	0.8708	0.8745
BLEU-2	0.7463	0.8555	0.8717
BLEU-3	0.7305	0.8412	0.8687
BLEU-4	0.7148	0.8278	0.8650
Semantic quality			
ROUGE-1	0.8239	0.9203	0.9223
ROUGE-2	0.7921	0.8981	0.9154
ROUGE-L	0.8203	0.9198	0.9203
METEOR	0.7630	0.8811	0.8688
Clinical relevance			
CIDEr	1.3456	2.5424	5.6489
Jaccard similarity	0.8232	0.9034	0.9099

The most significant improvement was in the CIDEr score, which rose by 122% from the coattention approach (2.5424) to MedFusionT5 (5.6489) and by 320% compared to simple concatenation (1.3456). This metric's sensitivity to distinctive, clinically relevant n-grams indicates that unidirectional alignment allows the model to capture specialised medical terminology and concepts more effectively than both concatenation and coattention methods.

Although performance differences between MedFusionT5 and coattention were modest across several metrics (ranging from 0.3% to 3.6% on BLEU scores), the substantial gap in CIDEr suggests that unidirectional alignment may offer superior clinical precision. Unidirectional attention reduces the ‘noise’ of complex bidirectional interactions, enabling a more focused integration of visual information into text generation.

**Fig. 3:**
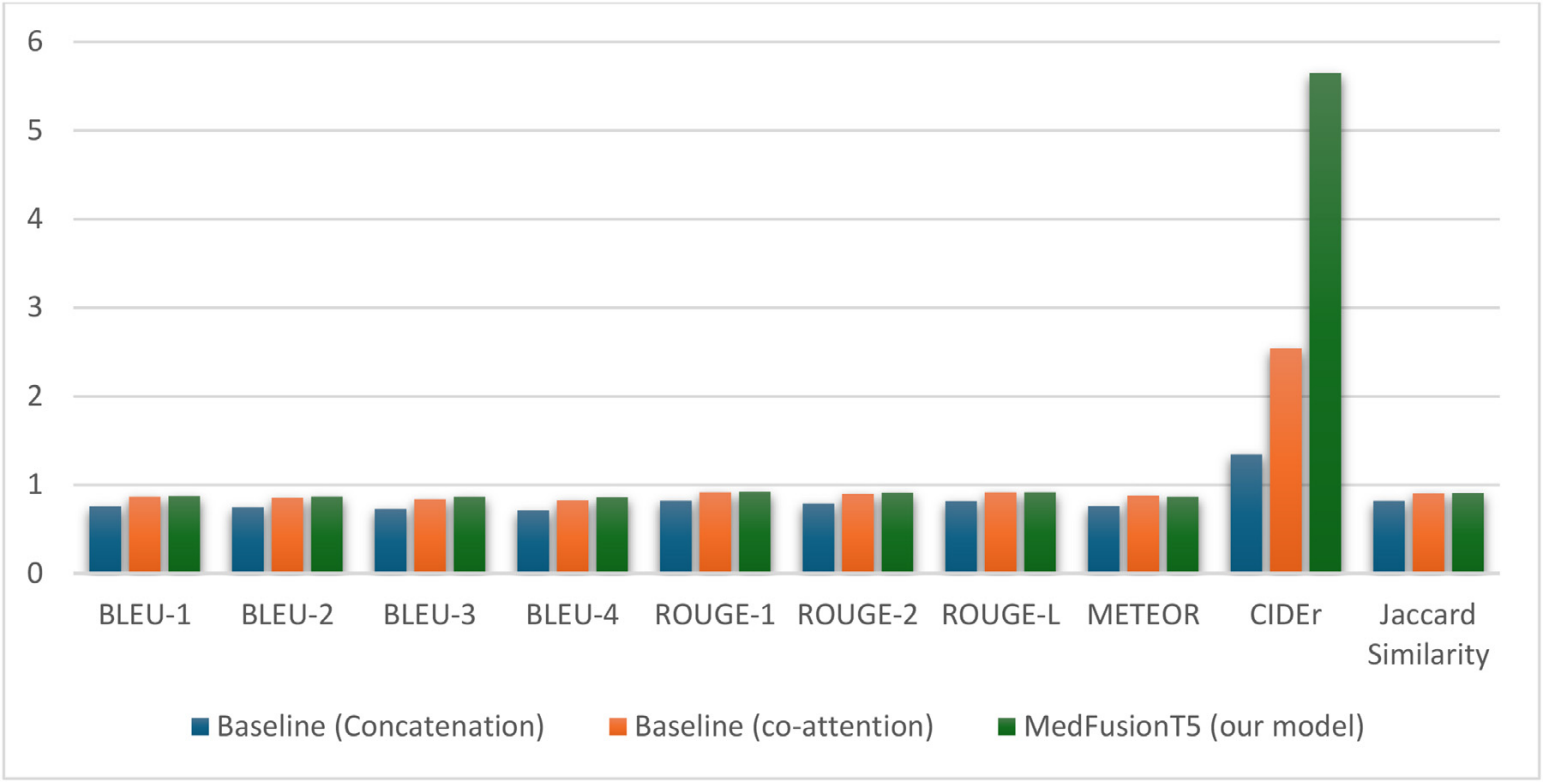
Unidirectional alignment (MedFusionT5) demonstrated superior performance, with CIDEr showing the most significant improvement (+122% over coattention and +320% over simple concatenation). BLEU, ROUGE, and Jaccard similarity metrics consistently outperformed both baselines, confirming that the benefits of unidirectional alignment extend to lexical precision, semantic adequacy, and clinical relevance, while minimising the noise associated with complex bidirectional interactions.

MedFusionT5 also achieved the best performance on 9 of 10 key metrics, with coattention only outperforming it on METEOR (0.8811 vs 0.8688). This suggests that while bidirectional attention may enhance synonym handling slightly, it comes at the cost of reduced clinical precision, as indicated by the significantly lower CIDEr score (Figure 3).

### Clinical efficacy analysis

Table 3 presents the token-level analysis focusing on clinical content capture.

**Table 3:** Clinical efficacy metrics.

Metric	Baseline (concatenation)	Baseline (coattention)	MedFusionT5 (our model)
Precision (Token)	0.9741	0.9796	0.9817
Recall	0.8411	0.9184	0.9234
F1 Score	0.9024	0.9465	0.9504

Compared to both baselines, MedFusionT5 shows consistent improvements across all token-level metrics. Against the concatenation baseline, recall improved substantially by 9.8%, indicating a significantly more comprehensive report generation. When compared to coattention, our approach achieved measurable gains in both precision (0.2%) and recall (0.5%), demonstrating progressive refinement. The F1 score improved by 5.3% over concatenation and by 0.4% over coattention, reflecting an optimal balance between completeness and correctness that is critical for clinical utility.

### Statistical significance

Table 4 presents the statistical validation results.

**Table 4:** Statistical significance testing.

Analysis	Baseline (concatenation)	Baseline (coattention)	MedFusionT5 (our model)
Paired t-test	t = −3.3514 *P* = .0021	t = −5.3512 *P* = .0000	t = −5.1336 *P* = .0000
Length-quality correlation	r = +0.3233	r = +0.7953	r = −0.0215

Paired t-tests confirmed that all models produced highly significant improvements over reference lengths (*P* < .01), with both attention-based approaches exhibiting even stronger significance (*P* < .0001). The correlation analysis reveals distinct behavioural patterns: coattention shows a strong positive correlation (r = +0.7953), indicating length-dependent quality, while our MedFusionT5 demonstrates a near-zero correlation (r = −0.0215), suggesting consistent quality regardless of output length. This implies that unidirectional alignment provides more stable generation quality across varying report complexities, in contrast to coattention's length-dependent performance.

### Hallucination analysis

The hallucination rate was computed automatically using a token-based approach that identifies novel content not found in the reference reports. We defined ‘unsupported content’ as unigrams and bigrams in the generated reports that are absent from the corresponding reference reports. The hallucination score for each report is calculated as a weighted combination of novel n-grams, with 60% assigned to unigrams and 40% to bigrams, relative to the total n-grams in the prediction. This automated method offers a consistent and reproducible measure of hallucination, capturing both isolated term errors and erroneous phrase constructions.

Table 5 presents the performance of models in controlling hallucinations.

**Table 5:** Hallucination analysis and qualitative metrics comparison across multimodal alignment strategies.

Analysis	Baseline (concatenation)	Baseline (coattention)	MedFusionT5 (our model)
Hallucination rate	0.0447	0.0337	0.0242
Hallucination control	0.9553	0.9663	0.9758
Semantic coherence	0.7660	0.8762	0.8847
Semantic accuracy	0.9059	0.9482	0.9519
Factual consistency	0.8236	0.9118	0.9161
Textual quality	0.5576	0.6693	0.7840

**Fig. 4:**
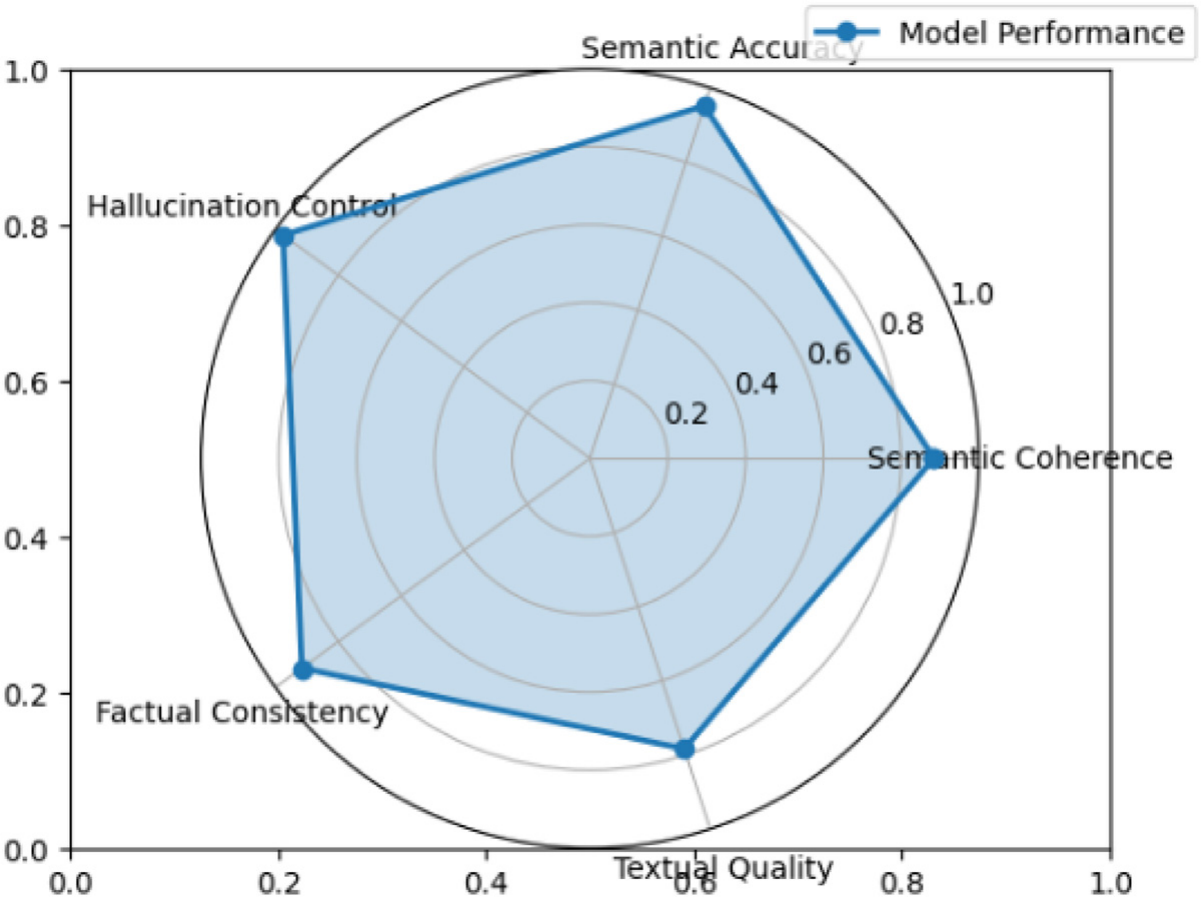
Qualitative evaluation of MedFusionT5. Our model exhibits balanced performance across all critical clinical metrics, achieving exceptional hallucination control (0.976) and semantic accuracy (0.952). The architecture also maintains strong factual consistency (0.916) while generating semantically coherent reports (0.885). These results affirm MedFusionT5′s reliability in medical report generation, effectively minimising erroneous content while ensuring clinical accuracy.

MedFusionT5 demonstrates superior performance in controlling hallucinations (Figure 4), achieving the lowest hallucination rate of 2.42%, compared to 3.37% for coattention and 4.47% for simple concatenation. This represents a significant reduction in hallucinations compared to the coattention approach and an improvement over the basic concatenation method. The consistent improvement across architectures clearly indicates that unidirectional alignment facilitates more controlled and factual generation, effectively minimising erroneous content while maintaining comprehensive reporting.

⮚ *Hallucination performance*

MedFusionT5 achieves the lowest hallucination rate at 2.42%, outperforming coattention at 3.37% and concatenation at 4.47%, respectively. This significant reduction demonstrates the superior control provided by unidirectional alignment in minimising erroneous content.

⮚ *Semantic and factual metrics*

MedFusionT5 excels in semantic coherence (0.885), accuracy (0.952), and factual consistency (0.916), surpassing both baselines. The improvement in factual consistency compared to concatenation confirms the method's effectiveness in preserving clinical integrity.

⮚ *Textual quality and clinical implications*

With a textual quality score of 0.784, 17% higher than coattention, MedFusionT5 generates more fluent and structured reports. Its low hallucination rate further enhances its suitability for clinical applications that require both accuracy and readability.

### Qualitative comparison

**Fig. 5:**
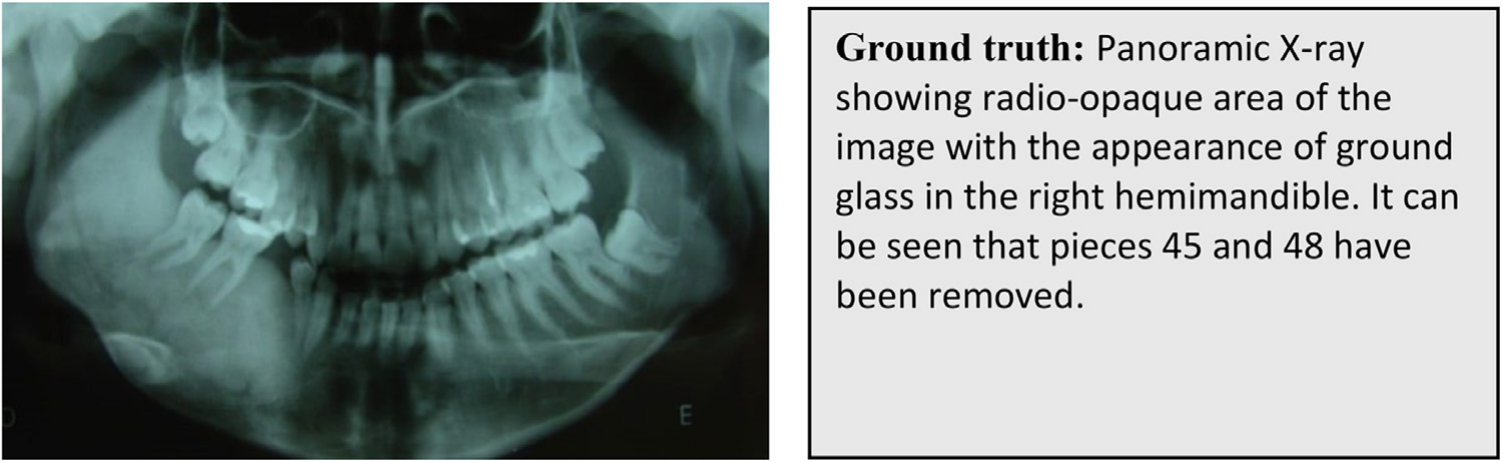
Example panoramic X-ray with corresponding generated reports.

MedFusionT5 generates the most precise clinical description with complete terminology (‘ground glass appearance’) and natural phrasing (‘missing’). Coattention produces accurate but slightly simplified reports, while concatenation shows significant quality degradation with informal language and omitted clinical details, validating our quantitative results (Figure 5 and Table 6).

**Table 6:** Qualitative comparison of generated medical reports across multimodal alignment strategies.

Baseline (Concatenation)	Baseline (coattention)	MedFusionT5 (our model)
Panoramic X-ray shows radiopaque ground glass in the right mandible. Teeth 45 and 48 are absent.	Panoramic X-ray shows a radiopaque area with ground glass in the right hemimandible. Teeth 45 and 48 are removed.	Panoramic X-ray shows radiopaque ground glass appearance in the right mandible. Teeth 45 and 48 are missing.

## Discussion

### Cross-modal alignment as performance catalyst

The 122% improvement in CIDEr over coattention and the 320% gain over concatenation signify a substantial advancement in medical report generation, where typical annual improvements in established benchmarks range from 2% to 5%.^62^ This significant leap validates our hypothesis that unidirectional alignment offers superior multimodal fusion compared to bidirectional approaches. CIDEr's sensitivity to distinctive, clinically relevant n-grams suggests that this improvement represents a genuine enhancement in capturing specialised medical terminology rather than merely optimising the metric.

The consistent BLEU improvements across all n-gram orders (ranging from 0.3% to 3.6% over coattention) indicate that the benefits of unidirectional alignment extend from lexical precision to phrase-level coherence. Higher-order n-grams require accurate terminology with proper clinical sequencing, confirming that our approach enhances both medical content selection and clinical linguistic quality.

These findings align with recent medical vision-language research that emphasises the importance of structured multimodal integration. Chen et al^63^ demonstrated that memory-enhanced cross-modal networks improve the completeness of radiology reports, while Zhou et al^64^ showed that knowledge-enhanced pretraining boosts clinical accuracy. Our work extends these approaches by demonstrating that unidirectional attention offers more effective alignment than bidirectional mechanisms in medical domains. Additionally, Zhang et al^65^ recently highlighted the challenges of multimodal hallucination in clinical settings, reinforcing our finding that focused alignment reduces erroneous generation. The combination of domain-specific encoders (ViT for medical imaging and Bio_ClinicalBERT for clinical text) with controlled unidirectional alignment proves particularly effective in addressing the precise terminology and factual consistency requirements of medical reporting.

### Balanced optimisation across metrics

The improvement in precision (BLEU) and recall (ROUGE) metrics shows holistic optimisation, not trade-offs. Our token-level F1 analysis confirmed this: substantial recall gains (+9.8% from the concatenation baseline) with maintained precision (+0.2% over coattention) indicate that MedFusionT5 generates more comprehensive reports without sacrificing accuracy.

This balance is clinically significant. High precision without adequate recall risks missing crucial findings, as shown in recent studies on multimodal medical AI,^66^ while excessive recall with poor precision produces verbose reports that can overwhelm clinicians.^67^ MedFusionT5′s F1 score of 0.9504 suggests it achieves the optimal balance for clinical utility.

The near-zero length-quality correlation (r = −0.0215) supports this interpretation. Unlike coattention's strong length-dependent performance (r = +0.7953), our unidirectional alignment maintains consistent quality regardless of report length. This addresses a key challenge in medical vision-language research, where models often struggle to maintain factual consistency across varying report lengths.^68^

### Hallucination mitigation through visual grounding

Our analysis shows that explicit visual-textual alignment effectively constrains model generations to clinically supported content. The architecture's unidirectional attention ensures that textual generation continuously references visual evidence, reducing unsupported claims while maintaining coverage. This approach addresses a key challenge in medical AI, where models must balance completeness with reliability.

The 2.4% hallucination rate achieved by MedFusionT5 represents a significant improvement over existing methods, especially considering recent findings on hallucination rates in medical LLMs. Studies indicate that specialised clinical models can have hallucination rates exceeding 15-20% when generating detailed medical descriptions.^69^^,^^70^ Our method's ability to maintain this low rate while enhancing report quality demonstrates the effectiveness of structured visual grounding for clinical use.

Recent research in medical vision-language models emphasises the importance of reliable generation for clinical deployment.^71^^,^^72^ The reduction in hallucinations across our architectural comparisons, from 4.5% with simple concatenation to 2.4% with unidirectional alignment, validates that explicit cross-modal interaction provides essential safeguards against unsupported content generation. This is especially critical for radiology reporting, where even minor inaccuracies can affect diagnostic decisions***.***

### Practical implications across health care settings

The performance improvements demonstrated by MedFusionT5 highlight its potential to support diagnostic workflows across a wide range of clinical environments. In rural hospitals, where access to on-site dental radiologists is often limited, automated preliminary reports can equip general practitioners with timely guidance while awaiting specialist confirmation.^3^ In acute care settings, such as emergency departments, rapid AI-generated interpretations may help clinicians prioritise trauma cases requiring immediate intervention.^4^^,^^5^ Similarly, military medical units deployed in remote or resource-limited environments could benefit from immediate diagnostic support when specialist expertise is unavailable.^6^ Telemedicine networks serving underserved communities may also leverage automated preliminary reads to mitigate the delays inherent in asynchronous teleradiology services, reducing turnaround times from days to minutes.^9^^,^^10^ After-hours facilities, where imaging is frequently performed without real-time specialist coverage, could likewise rely on MedFusionT5 to provide *interim* assessments that improve continuity of care until formal reports become available.^11^^,^^12^ Beyond these hospital-based applications, the model offers particular utility in sports medicine. Teams competing internationally or training in remote locations frequently encounter dental injuries that require immediate evaluation. With portable radiographic equipment, athletic trainers and team physicians could obtain near-instant interpretations to determine whether an athlete can safely continue participation or requires urgent referral.^16^

Together, these scenarios illustrate how MedFusionT5 may enhance diagnostic access and efficiency across heterogeneous health care systems.^7^^,^^8^ While human oversight remains essential, especially in complex cases, the model’s ability to deliver rapid, clinically coherent preliminary reports positions it as a valuable adjunct in settings constrained by workforce shortages, geographic barriers, or time-sensitive decision-making.

### Architectural implications

These results challenge the predominant paradigm of simple feature concatenation in multimodal medical AI. Our findings suggest that explicit interaction mechanisms, particularly cross-attention, are fundamental architectural requirements for high-quality medical report generation, rather than merely incremental refinements.

The success of attention-based alignment likely stems from its ability to model fine-grained correspondences between specific anatomical regions and clinical descriptions. Concatenation provides the decoder with both modalities but leaves the discovery of these correspondences to the decoding process itself. Alignment explicitly computes these correspondences before decoding, providing the generator with pre-aligned representations that naturally support coherent and grounded text production.

This principle likely extends beyond the field of dental radiology. Similar visual-textual grounding challenges exist across medical imaging modalities, including chest X-rays, CT scans, and MRI, suggesting that our architectural approach may generalise broadly.^35^^,^^36^ Future studies should validate MedFusionT5′s performance in diverse radiological domains to assess its transferability.

### Comparison with related work

A comprehensive comparison with recent state-of-the-art medical report generation methods highlights both the competitive performance of our MedFusionT5 framework and the fundamental challenges in cross-study evaluation. As summarised in Table 7, our method outperforms existing approaches across all standard natural language generation metrics. However, this comparison should be interpreted with caution due to significant methodological and dataset differences that limit direct comparability.

**Table 7:** Performance comparison with state-of-the-art medical report generation methods.

Method	Architecture	Dataset	BLEU-1	BLEU-2	BLEU-3	BLEU-4	ROUGE-L	METEOR	CIDEr
R2GenCSR^37^	Mamba + LLM + Context Retrieval	IU-X-ray	0.420	0.268	0.186	0.136	0.291	0.167	0.267
Cross-modal augmented transformer^38^	Prealignment + Dual-stream Decoder	IU-X-ray	0.491	0.327	0.233	0.176	0.383	0.195	0.457
MedVAG^73^	ViT + GPT-2 + multiattention	IU-X-ray	0.761	0.691	0.642	0.595	0.762	0.748	5.054
		COV-CTR	0.808	0.738	0.674	0.611	0.805	0.807	2.226
MedFusionT5 (Ours)	ViT + BioClinicalBERT + multihead cross-attention	Dental panoramic	0.875	0.872	0.869	0.865	0.920	0.869	5.649

The comparative analysis shows that MedFusionT5 achieves state-of-the-art performance across all evaluated metrics, with especially notable advantages in BLEU scores (BLEU-4: 0.865 vs a maximum of 0.611 for comparative methods) and ROUGE-L (0.920 vs a maximum of 0.805). Additionally, the CIDEr score of 5.649 surpasses the best comparative result of 5.054 achieved by MedVAG on the IU X-Ray dataset.

However, a fundamental limitation of this analysis lies in the disparities between domains and datasets. While MedVAG^73^ is the closest competitor in terms of architectural sophistication and performance, its evaluation on chest X-ray and CT datasets involves significantly different clinical content and reporting conventions compared to dental panoramic radiography. The anatomical complexity, terminology specificity, and structural patterns in dental imaging present unique challenges that may not be directly comparable to thoracic radiology reporting.

Moreover, the scarcity of public panoramic dental datasets poses a significant constraint, necessitating the use of a specialised collection, unlike the established chest X-ray benchmarks (IU-Xray, MIMIC-CXR) employed by other researchers. This dataset disparity highlights the critical need for standardised evaluation frameworks in medical report generation, particularly for specialised domains like dental radiology, where public benchmarks remain limited.

The performance advantage demonstrated by MedFusionT5 can be attributed to our focused unidirectional alignment strategy, which appears particularly effective for capturing the precise anatomical relationships and terminology specificity required in dental reporting. Future work should address these comparative limitations through cross-domain evaluation initiatives and the development of standardised multi-domain medical reporting benchmarks to enable more rigorous methodological comparisons.

### Limitations and future directions

This study has several limitations that warrant consideration. Our evaluation was based on a limited dataset of 700 panoramic radiographs from a single source, which may restrict the generalizability of the model across diverse health care settings and imaging protocols. The lack of large-scale public panoramic X-ray datasets poses significant validation challenges, as models may learn institution-specific biases instead of generalizable medical knowledge.^74^

Future work should focus on multi-institutional collaboration to create comprehensive benchmark datasets that capture the full spectrum of clinical variability in panoramic imaging. External validation across diverse health care settings is essential to verify the model's robustness before clinical deployment. Additionally, prospective studies that assess real-world clinical impact will be crucial for translating these technical advances into improved patient care.

Second, while we measured hallucination rates, our method relied on text comparison rather than expert clinical review. More rigorous validation would involve radiologists assessing whether the generated reports accurately reflect the image content, potentially revealing hallucinations that our automated method missed. Third, prospective clinical trials comparing MedFusionT5 reports against radiologist interpretations would provide definitive evidence of clinical utility.^75^

Fourth, our focus on dental radiology, while addressing an important clinical need, represents a relatively constrained area. Extending to more complex imaging modalities, such as 3D CT or multiplanar MRI, with longer, more complex reports would test architectural scalability and generalization.^76^

Fifth, we did not investigate the real-time performance or computational efficiency. Clinical deployment requires an acceptable inference speed and resource requirements, in addition to accuracy. The efficiency profile of our current implementation requires systematic characterisation, including inference time, memory footprint, and energy consumption.^77^

Finally, uncertainty quantification is an important direction for future research. Enabling the model to flag low-confidence generations would further enhance clinical trustworthiness by alerting clinicians to cases requiring careful review.^78^ Integrating Bayesian approaches or ensemble methods could provide confidence estimates for each generated report.

Future research should address these limitations through multi-institutional validation, expert clinical evaluation, cross-modality testing, performance optimisation, and uncertainty quantification. Additionally, investigating hierarchical or graph-based alignment mechanisms could provide stronger visual-textual grounding for complex cases.^79^

## Conclusion

We introduced MedFusionT5, a unidirectional alignment architecture for automated dental radiology report generation that establishes new performance standards through the structured integration of visual and textual modalities. Our approach demonstrates that unidirectional attention between visual features (ViT) and clinical embeddings (Bio_ClinicalBERT) simultaneously enhances semantic quality, achieving a 320% improvement in CIDEr and a 17% improvement in BLEU-4 over concatenation, while maintaining strong factual grounding with a 2.42% hallucination rate, the lowest among the three architectures.

These results position unidirectional alignment as a crucial architectural component for medical report generation, outperforming both simple concatenation and bidirectional coattention approaches. The performance gains observed across all metrics (lexical precision, semantic adequacy, and clinical completeness), combined with superior hallucination control, address fundamental limitations that have hindered the clinical adoption of automated systems.

However, our study is limited by the size and diversity of our dataset. The scarcity of public dental panoramic radiography databases, comprising only 700 images from a single source, presents significant challenges for validation and generalisation. Performance could vary substantially with images from different institutions using varied acquisition protocols.

The practical implications of this research extend to various clinical contexts where access to radiological expertise is limited. While human validation remains essential, automated preliminary report generation could enhance diagnostic access in underserved areas. Future studies should prioritise the collection of larger multi-institutional datasets and prospective validations to confirm the clinical robustness of our approach. In conclusion, MedFusionT5 represents a significant advance toward medical AI systems that combine clinical utility and reliability, but its clinical deployment will require rigorous validation on data that is more representative of real clinical diversity.

## Author contributions

*Responsible for the methodology design, data curation, software implementation, formal analysis, and visualisation, and prepared the original draft of the manuscript*: Abdaoui.

*Contributed to the investigation, resource provision, validation, and manuscript review and editing:* Dergaa, Barbaria, Ceylan, Bragazzi, de Giorgio.

*Participated in the conceptualisation and literature review, provided supervision, and contributed to the final review and editing of the manuscript*: Rahmouni.

*Contributed to the conceptualisation and literature review, provided oversight throughout the process, and contributed to the final review and editing of the manuscript:* Salah

*Read and approved the final version of the manuscript and agree to be accountable for all aspects of the work:* All authors.

## Ethics statement

This study was conducted in accordance with the Declaration of Helsinki (revised in 2013). The dataset was obtained from the OpenI repository (https://openi.nlm.nih.gov/), a publicly accessible resource provided by the U.S. National Library of Medicine. All images and reports in this repository are fully de-identified and anonymised to protect patient privacy. Therefore, no additional Institutional Review Board (IRB) approval was required for using this publicly available dataset.

## Declaration of generative AI and AI-assisted technologies in the writing process

In preparing this manuscript, the authors used ChatGPT 5 on September 15, 2025, to refine the language clarity, improve the academic English, and check for grammatical errors. After using this tool, the authors reviewed and edited all the content and took full responsibility for the publication.

## Funding

This research did not receive any specific grants from any funding agencies in the public, commercial, or not-for-profit sectors.

## Conflict of interest

None disclosed.
